# Variants of *GSK3β* and *SFRP4* genes in Wnt signaling were not associated with osteonecrosis of the femoral head

**DOI:** 10.18632/oncotarget.20393

**Published:** 2017-08-22

**Authors:** Yang Song, Zhenwu Du, Qiwei Yang, Ming Ren, Qingyu Wang, Gaoyang Chen, Haiyue Zhao, Zhaoyan Li, Guizhen Zhang

**Affiliations:** ^1^ Department of Orthopedics of Second Clinical College of Jilin University, Changchun, 130041, China; ^2^ Research Centre of Second Clinical College of Jilin University, Changchun, 130041, China; ^3^ The Engineering Research Centre of Molecular Diagnosis and Cell Treatment for Metabolic Bone Diseases of Jilin Province, Changchun, 130041, China

**Keywords:** ONFH, SFRP4, GSK3β, gene variants, Wnt signaling

## Abstract

Genome-wide association studies have identified that the gene variants in Wnt signaling associate with bone mineral density and fracture risk but the effects of the variants on the development of osteonecrosis of the femoral head (ONFH) have been unclear. Here, we analyzed the polymorphisms of 4 variants in *GSK3β* and *SFRP4* genes of Wnt signaling and their association with the development of ONFH through Mass ARRAY^®^ platform in 200 ONFH patients and 177controls in Chinese population. Our results showed that the genotypes and allele frequencies of all variants detected in *SFRP4* and *GSK3β* genes were not significantly different between patients and controls (*p* > 0.05); the correlation analysis between the 4 variants genotypes and gender, age at onset, etiological classification, unilateral or bilateral hip lesions, and clinical stages of ONFH, respectively, did not confirm significant association (*p* > 0.05) although age at onset in the minor homozygous(CC) carriers of *SFRP4* rs1052981 (T/C) was a statistically younger tendency than that of the major homozygous (TT) or heterozygous (TC) of the SNP (*p* = 0.051); moreover, all haplotypes analyzed and their association with the clinical phenotypes of ONFH were also shown no statistical significance (*p* > 0.05).These results suggest that the 4 variants analyzed by this study in *GSK3β* and *SFRP4* genes of Wnt signaling pathway are unlikely to be associated with susceptibility to ONFH.

## INTRODUCTION

Wnt signaling pathway, as a crucial regulator of tissue homeostasis and remodeling, plays key roles in the transdifferentiation between osteogenesis and adipogenesis of bone marrow mesenchymal stem cells (BMMSCs) [1, 2].The Wnt family is comprised of 19 secreted cysteine-rich glycoproteins. Depending on binding to one of the ten different Frizzled receptors and other coreceptors on the cell surface, they activate canonical, noncanonical, or both pathways for transcription of target genes [3]. The canonical Wnt signaling has been implicated in stimulating osteoprogenitor proliferation and osteogenesis [4]. The Wnt signaling represses adipogenesis by blocking the induction of CCAAT/enhance-binding protein-α (CEBPA) and peroxisome proliferator-activated receptor-γ (PPARγ), two master adipogenic transcription factors, and the disruption of Wnt/β-catenin signaling leads to spontaneous adipogenesis [5].

Osteonecrosis of the femoral head (ONFH) is a complex disease caused by the interaction with the genetic and environmental factors [6]. Multiple gene variants have been proposed as the genetic risk factors of ONFH [7] but its molecular pathogenesis has been remained obscure. ONFH prevalence has been increased in recent decades [8]. Genome-wide association studies (GWAS) have identified that common variants of genes in Wnt signaling associated closely with bone mineral density (BMD) and risk of fracture, and several recent GWAS studies demonstrate that genetic variations in Wnt16 are correlated with BMD and risk of fracture in children and adults across multiple populations [9–11]. However, it has never been reported that the gene variants in the Wnt signaling associate with the development of ONFH. Here, we analyzed the genotypes, allele, haplotype frequencies of 4 variants of glycogen synthase kinase 3 beta (*GSK3*β) and secreted frizzled-related protein 4 (*SFRP4*) genes in Wnt signaling and their association with the risk and clinical phenotypes of ONFH in 200 ONFH patients and 177controls in Chinese population.

## RESULTS

### Genotypes and allele frequencies of 4 variants in the *SFRP4* and *GSK3β* genes between ONFH and control groups

Genotypes and allele frequencies of 4 variants in the *SFRP4* and *GSK3β* genes are shown in Table 1. X^2^ test results showed that the genotypes of S*FRP4* rs1052981 (C/T) and rs1802073 (A/C) between ONFH and control groups were no statistical significance, *p* = 0.161, *p* = 0.494, respectively; logistic regression analyses further revealed that all models in the two variants, including codominant, dominant, and recessive, failed to show statistical significance (*p* > 0.05); their allele frequencies were also no statistical difference between ONFH and control groups, *p* = 0.202, *p* = 0.351, respectively. The genotypes of GSK3β rs3732361 (A/G) and rs3755557 (A/T) between ONFH and groups showed no statistical significance, *p* = 0.275, *p* = 0.622, respectively, and their allele frequencies were also not statistically different between ONFH and control groups, *p* = 0.938, *p* = 0.830, respectively.

**Table 1:** Genotype and allele frequencies of the variants in the GSK3B and SFRP4 between ONFH patients and controls

Gene	SNP ID	Group	Genotype (n)			MAF	HWE^a^	*p*^b^	Co-dominants (11 vs. 12 vs. 22)	Dominants 12+22 vs. 11	Recessives 22 vs. 11+12	Allele 2 vs. 1
			11	12	22				OR (95%CI) *P*^c^	OR (95%CI) *P*^c^	OR (95%CI) *P*^c^	OR (95%CI) *P*^c^
GSK3β	rs3732361 (A/G)		AA	AG	GG							
		Control	51	94	31	0.443	0.276	0.275	1.036 (0.760–1.412)	0.882 (0.549–1.416)	1.315 (0.763–2.267)	0.988 (0.7311–1.335)
		Case	55	75	37	0.446	0.238		0.824	0.602	0.324	0.938
	rs3755557 (T/A)		TT	AT	AA							
		Control	130	42	4	0.142	0.781	0.622	0.971 (0.632–1.491)	1.028 (0.640–1.649)	0.462 (0.083–2.578)	0.955 (0.630–1.449)
		Case	143	49	2	0.137	0.324		0.892	0.910	0.379	0.830
SFRP4	rs1052981 (T/C)		TT	CT	CC							
		Control	96	68	13	0.266	0.841	0.161	0.824 (0.572–1.188)	0.878 (0.569–1.355)	0.457 (0.157–1.329)	0.799 (0.566–1.128)
		Case	101	68	5	0.224	0.103		0.299	0.577	0.151	0.202
	rs1802073 (A/C)		AA	AC	CC							
		Control	47	87	43	0.489	0.827	0.494	1.106 (0.820–1.493)	1.330 (0.814–2.174)	0.985 (0.607–1.597)	0.872 (0.654–1.163)
		Case	42	104	51	0.477	0.415		0.510	0.254	0.950	0.351

11: homozygotes for the major allele, 12: heterozygotes and 22: homozygotes for the minor allele.

^a^*p*-values of deviation from Hardy-Weinberg equilibrium between the ONFH group and control group.

^b^χ^2^ test were used for frequencies of genotype analyses

^c^Logistic regression analyses were used for calculations. bold: *p*-value < 0.05

### The association of 4 variants genotypes in the *SFRP4* and *GSK3β* genes with the clinical phenotypes of ONFH

We completed the correlation analysis between the 4 variants genotypes of *SFRP4*, *GSK3β* genes and gender, age at onset, etiological classification, unilateral or bilateral hip lesions, and clinical stages of ONFH, respectively. The results confirmed that all genotypes were not shown the statistical association with the clinical phenotypes of ONFH although age onset in the minor homozygous (CC) genotype carriers of *SFRP4* rs1052981 (T/C) revealed statistically younger tendency than that of the major homozygous (TT) or heterozygous (TC) of the SNP (*P* = 0.051) and the male proportion of heterozygous (TC) of *GSK3β* rs3755557 revealed a increased statistical tendency compared with the female proportion of the TC genotype, *P* = 0.067, presented in Table 2.

**Table 2:** The association of genotypes in the 4 variants of SFRP4 and GSK3β genes with clinical phenotypes of ONFH

Gene	SNP IP	Genotype	Gender *n* (%)		Age on set (yr) ANOWY	Etiological classification *n* (%)▲			Hip lesions *n* (%)▲		Clinical stages *n* (%)▲		
			Male	Female		Alc^a^	Ster	Idio	Unilateral	Bilateral	StageII	StageIII	StageIII
SFRP4	rs1802073 (C/A)	CC	29 (24.6)	15 (25.9)	46.9±13.5	16 (22.9)	12 (26.7)	16 (26.2)	19 (25.0)	25 (25.0)	3 (30.0)	16 (30.2)	25 (22.3)
		CA	67 (56.8)	28 (48.2)	48.8±12.1	37 (52.9)	23 (51.1)	35 (57.4)	44 (57.9)	51 (51.0)	5 (50.0)	26 (49.1)	64 (56.6)
		AA	22 (18.6)	15 (25.9)	46.9±13.5	17 (24.3)	10 (22.2)	10 (16.4)	13 (17.1)	24 (24.0)	2 (20.0)	11 (20.7)	24 (21.2)
		*P*	0.470		0.185	0.838			0.507		0.832		
	rs1052981 (T/C)	TT	63 (59.4)	27 (54.0)	46.0±11.9	33 (55.0)	24 (57.1)	33 (61.1)	36 (57.1)	54 (58.1)	4 (4.0)	28 (62.2)	58 (57.4)
		TC	40 (37.7)	22 (44.0)	49.6±11.9	25 (41.7)	16 (38.1)	21 (38.9)	27 (42.9)	35 (37.6)	6 (60.0)	16 (35.6)	40 (39.6)
		CC	3 (2.8)	1 (2.0)	36.7±9.8	2 (3.3)	2 (4.8)	0 (0.0)	0 (0)	4 (4.3)	0 (0)	1 (2.2)	3 (3.0)
		*P*	0.741		**0.051**	0.628			0.326		0.685		
GSK3β	rs3732361 (A/G)	AA	35 (33.7)	12 (25.5)	46.0±12.1	20 (34.5)	16 (39.0)	11 (21.2)	22 (36.7)	25 (27.5)	2 (20.0)	15 (34.1)	30 (30.9)
		AG	45 (43.3)	25 (53.2)	48.1±13.2	22 (37.9)	17 (41.5)	31 (59.6)	27 (45.0)	43 (47.3)	3 (30.0)	18 (40.9)	49 (50.5)
		GG	24 (23.1)	10 (21.3)	48.5±11.8	16 (27.6)	8 (19.5)	10 (19.2)	11 (18.3)	23 (25.3)	5 (50.0)	11 (25.0)	18 (18.6)
		*P*	0.492		0.587	0.145			0.408		0.207		
	rs3755557 (T/A)	TT	83 (69.7)	41 (74.5)	48.4±11.7	49 (71.0)	35 (74.5)	40 (69.0)	0 (0)	2 (2.0)	8 (80.0)	38 (71.7)	78 (70.3)
		TA	36 (30.3)	12 (21.8)	45.3±13.5	20 (29.0)	11 (23.4)	17 (29.3)	47 (65.3)	77 (75.5)	2 (20.0)	14 (26.4)	32 (28.8)
		AA	0 (0)	2 (3.6)	46.0±10.0	0 (0)	1 (2.1)	1 (1.7)	25 (34.7)	23 (25.5)	0 (0)	1 (1.9)	1 (0.99)
			**0.067**		0.326	0.759			0.111		0.932		

^a^:Alc : Alcohol-induced, Ster: steroid- induced;, Idio: idiopathic; ▲X^2^ text

### The haplotypes of 4 variants in the *SFRP4* and *GSK3β* genes and their association with the clinical phenotypes of ONFH

**Figure 1:**
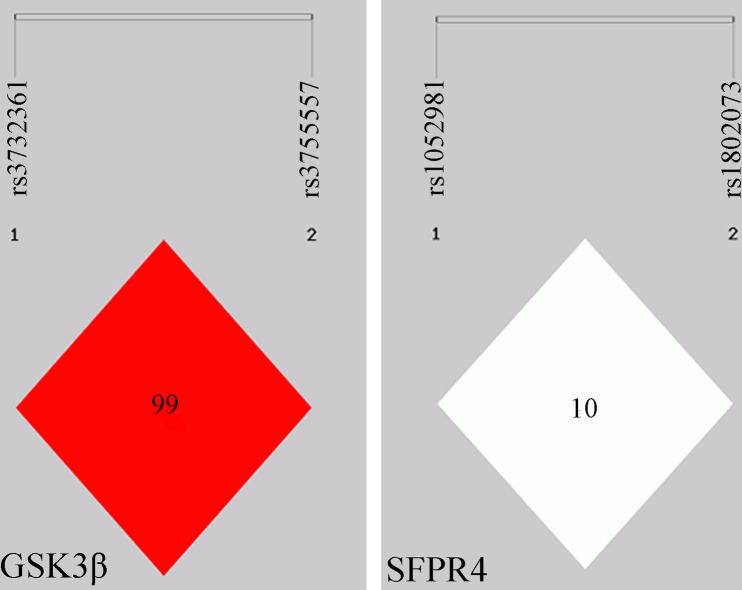
Linkage Disequilibrium (LD) map for the 4 variants in the GSK3β and SFRP4 genes Each diamond represents the correlation (D’) between each pair of variants with darker shades representing stronger linkage disequilibrium.

We calculated the Linkage disequilibrium (LD) coefficients between the 2 variants in the *SFRP4* and *GSK3β* genes, respectively, using Shesis software platform (http://analysis.bio-x.cn/SHEsisMain.html) on the basic of the variants genotypes, shown in Figure 1. LD analysis reveals a stronger LD between rs3732361 and rs3755557 of *GSK3β* gene. Usually, single tag SNP in the stronger LD enough captures the genetic information of correlated variants. However, considering the two variants located in different function (3′UTR or promoter) region might affect their potential association with ONFH, we still genotyped the two variants of *GSK3β* gene and the results demonstrated that the two variants in stronger LD did show no any association with the development of ONFH. The haplotypes analysis shows that there are the 4 haplotypes of C-A, C-C, T-A, and T-C between rs1052981(C/T) and rs1802073 of *SFRP4* gene and the 4 haplotypes were not shown significant difference between ONFH and control groups (*p* > 0.05) in spite of the decreased C-A haplotype frequency tendency of ONFH group, compared with control group, *P* = 0.073. There are the 4 haplotypes of A-A, A-T, G-A, and G-T between rs3732361 (A/G) and rs3755557 (A/T) of *GSK3β* gene, and the haplotypes were not statistically different between ONFH and control groups (*P* > 0.05), shown in Table 3. The haplotypes of *SFRP4* and *GSK3β* genes did not show significant association with the hip lesions of ONFH (*P* > 0.05), shown in Table 4.

**Table 3:** Haplotype analysis of GSK3β and SFRP4 genes in WNT signaling pathway

SNPs	Haplo-type	ONFH n (%)	Control n (%)	χ2	*P*	OR (95%CI)
SFRP4	C-A	37.91(11.0)	55.35(15.6)	3.210	**0.0732**	0.668 (0.429∼1.041)
rs1052981(C/T)-	C-C	39.09(11.4)	38.65(10.9)	0.035	0.8517	1.046 (0.653∼1.676)
rs1802073(A/C)	T-A	128.09(37.2)	125.65(35.5)	0.229	0.6326	1.078 (0.792∼1.468)
	T-C	138.91(40.4)	134.35(38.0)	0.432	0.5109	1.107 (0.817∼1.501)
GSK3β	A-A	45.99(13.8)	49.99(14.2)	0.026	0.871	0.965 (0.627∼1.486)
rs3732361 (A/G)-	A-T	139.01(41.6)	146.01(41.5)	0.001	0.971	1.006 (0.742∼1.363)
rs3755557 (A/T)	G-T	148.99(44.6)	155.99(44.3)	0.006	0.938	1.012 (0.749∼1.368)
	G-A	0.01(0.0)	0.01(0.0)	-	-	-

**Table 4:** Association of haplotypes of SFRP4 and GSK3β genes with the hip lesions of ONFH

SNPs	Haplo-type	Bilateral *n* (%)	Unilateral *n* (%)	χ2	*P*	OR (95%CI)
SFRP4	A-C	23.64 (13.0)	10.68 (8.5)	1.53	0.22	1.612 (0.753∼3.453)
rs1802073(A/C)-	A-T	69.36 (36.7)	45.32 (36.0)	0.146	0.70	1.096 (0.684∼1.755)
rs1052981(C/T)	C-C	18.36 (10.1)	16.32 (13.0)	0.613	0.43	0.754 (0.371∼1.532)
	C- T	70.64 (38.8)	53.68 (42.6)	0.444	0.51	0.855(0.538∼1.357)
GSK3β	A-A	24.00 (13.2)	21.00 (17.5)	1.061	0.30	0.716 (0.379∼1.354)
rs3732361(G/A)-	A-T	69.00 (37.9)	50.00 (41.7)	0.427	0.51	0.855 (0.534∼1.368)
rs3755557(T/A)	G-T	89.00 (48.9)	49.00 (40.8)	1.897	0.17	1.387(0.870∼2.209)

## DISCUSSION

Wnt signaling functions, as a molecular switch, determine the balance between osteogenesis and adipogenesis of BMMSCs [12, 4]. BMMSCs are multipotent cells that can differentiate into adipocytes or osteocytes, and the functional changes of BMMSCs differentiation pathway may contribute to the pathogenesis of ONFH [13, 14]. Moreover, interactions associated with the differentiation pathway of BMMSCs that promote adipogenesis and repress osteogenesis are considered as the major factors leading to steroid-related ONFH [15]. BMMSCs may differentiate preferentially into adipocytes rather than osteoblasts during the development of ONFH [16]. In view of the key roles of Wnt signaling in the differentiation of BMMSCs, we analysed the association of 4 variants in the *SFRP4* and *GSK3β* genes of this pathway with the risk and clinical phenotypes of ONFH. Our results found that the genotypes, alleles, haplotypes frequencies of all detected variants in the *SFRP4* and *GSK3β* genes were not significantly different between ONFH and controls groups; in order to explore the effects of the variants on the development of ONFH, we further completed the correlation analysis between the 4 variants genotypes and gender, age at onset, etiological classification, unilateral or bilateral hip lesions, and clinical stages of ONFH, respectively. These results further confirmed that all variants analyzed did not show statistical association with clinical phenotypes of ONFH, which suggests that the variants detected in this study are unlikely to be associated with susceptibility to ONFH.

SFRP4 is a member of the SFRP family that contains a cysteine-rich domain homologous to the putative Wnt-binding site of frizzled proteins. SFRPs act as soluble modulators of Wnt signaling, and SFRP4 has been identified as a molecular link between islet inflammation and defective insulin secretion. SFRP4 influences a wide scope of genes of Wnt signaling and several genes variants from the pathway have been linked to the pathogenesis of Type 2 diabetes mellitus [17]. SFRP4 is secreted by multiple tissues including adipose tissue, which contributes the elevated circulating SFRP4 level in obesity [18]. The genetic studies provided evidence that recessive mutations in *SFRP4* are a cause of Pyle's disease, and the mouse model in *SFRP4* mutations reproduced the human phenotype to a remarkable degree, which showed that the phenotype was due to the differential regulation of Wnt signaling and BMP signaling. Deletion of *SFRP4* activated predominantly canonical Wnt signaling in trabecular bone, leading to increased trabecular bone mass [19]. A study to detect the differentially expressed genes (DEGs) between ossified herniated discs and herniated discs without ossification showed that three of the top 20 DEGs, including sclerostin, WNT inhibitory factor 1, and *SFRP4* etc were correlated with the inhibition of Wnt pathway, which suggested that Wnt pathway abnormality and local inflammation may be correlated with disc ossification [20].

The protein encoded by *GSK3β* gene is a serine-threonine kinase, belonging to the glycogen synthase kinase subfamily. It involved in energy metabolism, neuronal cell development, and body pattern formation. Variants of *GSK3β* gene have been implicated in relating to risk of Parkinson disease (PD), and studies in mice show that overexpression of this gene may be relevant to the pathogenesis of Alzheimer's disease. GSK3, as an enzyme of intracellular signaling and metabolic control of the cell, is among the molecular constraints which keep chondrocytes in the “arrested state” [21], and it belongs to the β-catenin degradation complex and acts by keeping an inactive phosphorylated form of β-catenin thus preventing its nuclear translocation and transcriptional activation of lymphoid enhancer factor/T cell factor transcription factors (LEF/TCF) complex. A tightly regulated level of β-catenin signaling must be guaranteed for a healthy articular cartilage [22].

Previous studies have demonstrated that human osteoarthritis (OA) tissues over-express smad ubiquitin regulatory factor2 (Smurf2), whose conditional over-expression in mice is followed by inhibition and proteasomal degradation of GSK3β, upregulation of β-catenin, and articular cartilage degeneration [23, 24]. Metabolic Syndrome (MetS) is a global epidemic, affecting 23% of the general population with more than 2.5 fold prevalence in OA patients and indeed greatly worsen the risk of occurrence and progression of knee OA [25, 26]. A study for the extent of GSK3β inactivation in OA knee cartilage explants found occurrence of articular chondrocytes with inactive GSK3β in obese patients thus hinting at GSK3β as one potential mechanism whereby metabolic factors impact on OA [27]. A Greek study was the first to show that *GSK3*βrs334558 was related to PD, homozygous CC served a protective effect in PD [28], and a result from Australian population revealed that homozygous TT frequency of this SNP in PD patients was significantly increased compared to control subjects [29] while a result from Chinese population did not find any significant difference in allele-wise and genotype-wise analysis for GSK3βrs334558 between PD patients and controls.

To our knowledge, there were no report of an association of *SFRP4* and *GSK3*βpolymorphisms with the development of ONFH. We speculated that SFRP4 and GSK3β,as crucial proteins of Wnt signaling, might exert an important effect on the development of ONFH, and their polymorphisms might involved in ONFH risk. Therefore, we selected *SFRP4* and *GSK3*βgenes as potential candidate genes for susceptibility to ONFH. Our results demonstrated that the genotypes, allele, haplotype frequencies of 4 variants detected in the *GSK3*βand *SFRP4* genes and their association with the risk and clinical phenotypes of ONFH did not show statistical significance between 200 ONFH patients and 177controls. Nevertheless, the association of the variants in the *SFRP4* and *GSK3*βgenes with the risk and development of ONFH need to be further investigated with larger cohort studies.

The genes and their variants selection are a crucial element for exploring the effects of gene variants on a complex disease. The most important consideration is that new genes and their variants need to be identified to maximise the potential for associations [30]. Our major concern was the variants in promoter, 3-UTR, and coding region with the consideration of potential effects of them on the gene expression and gene function. Generally, variants in promoter and 3-UTR regions potentially contribute to differential gene expression, presumably affecting the binding of transcription factors to DNA. Therefore, we selected the 1 variant in promoter and 3-UTR region of *GSK3β* gene, the 1 variant in 3-UTR region of SFRP4 gene, respectively as target variants. Moreover, in view of the possible effects of variants in exons on protein expression, function or activity, we also selected the 1variant in coding region of *SFRP4* gene, as target variant. In spite of the 4 variants detected in this study failing to show statistical association with ONFH risk, the optimal selection strategy will further improve the association investigation of the other variants in the *SFRP4* and *GSK3*β genes as well as the other genes of Wnt signaling pathway with the risk and development of ONFH.

Our study has some limitations. First, the 377 cohort study attributes to smaller samples system, which may limit our statistical power to detect small differences between ONFH and control groups, especially for the subgroups analysis between the genotypes and clinical phenotypes. Second, we failed to detect the gene expression duo to the samples limitation. In future study, it is very significant to analyse the effects of the variants on the gene expression in larger sample system to identify their roles in the development of ONFH.

In conclusion, our results were not found the evidence supporting the 4 variants of *SFRP4* and *GSK3*β genes associated with the risk and development of ONFH in Chinese population, which suggest that the variants analyzed in this study are unlikely to be associated with susceptibility to ONFH.

## MATERIALS AND METHODS

### Individuals

Unrelated ONFH patients (132 men, 68 women; age: 52.8 ±9.7 yr) and 177 control individuals (112 men, 65 women; age: 50.73 ±11.02 yr ) who visited The Department of Orthopedics from March, 2014 to June, 2015 and the Health Examination Centre of Second Clinical College of Jilin University, (Changchun, China) from October 2014 to December 2014 in the study, respectively. The ONFH patients concurrent with direct trauma, severe chronic diseases, such as cardiovascular diseases, congenital diseases, human immunodeficiency virus (HIV) infection, diabetes mellitus, renal dysfunction, and cancer were excluded. ONFH were diagnosed by evidence of osteonecrosis using plain radiographs in Stages 2, 3, and 4 of the Ficat Classification system [31]. According to etiological factors, ONFH patients were subgrouped into alcohol-induced (71 cases (39.7%), idiopathic (64 cases (34.0%), and steroid-induced osteonecrosis (47 cases (26.3%), respectively. Steroid-induced osteonecrosis was defined by a history of taking prednisolone cumulative 2000mg or an equivalent over 21 days. Alcohol-induced osteonecrosis was defined by the consumption of more than 900 ml of pure ethanol per week. The course of ONFH ranged from 0.5 months to 360 months, with an average of 71.75 months, and the clinical stages of ONFH consisted of 10 cases of stage II (5.6%), 54 cases of stage III (30.2%) and 115 cases (64.2%) of stage IV.

The unilateral and bilateral hips lesions were 76 cases (42.5%) and 103cases (57.5%), respectively. There were 21cases of ONFH patients who failed to undergo the clinical stages or aetiological classification duo to the defect of plain radiographs or unclear aetiological factors. Health control subjects were defined in the same way as reference [32]. All of the 377 participants were Han Chinese from northeast China. The study was approved by the ethics committee of the Second Clinical College of Jilin University, Changchun, China, and conformed to the current ethical principles of the Declaration of Helsinki. All participants provided informed consent for their taking part in the study.

### Genomic DNA extraction and variants selection

Genomic DNA was extracted from whole blood samples using the genomic DNA extraction kit (DP318, TianGen, BeiJing, China) according to the manufacturer's protocols. The HapMap database and related literature were used to select variants of the genes by analysing their population distribution in different countries, nationalities and regions, particularly in data from an Asian population. The database: http://gvs.gs.washington.edu /GVS138/ was used to select variants. The search scope of the genes was from the upstream 2000bp to downstream 1000bp of *GSK3*β and *SFRP4* genes, respectively. The selection criteria of variants included in r^2^ > 0.8 or D’ = 1; Minority allele A frequencies > 0.05. Rs1802073 (T/G) in coding region and rs1052981 (G/A) in 3-UTR region of SFRP4 gene, rs3732361 (G/A) in 3-UTR region and rs3755557 (A/T) in promoter region of GSK3β gene were selected, shown in Table 5.

**Table 5:** Basic information of SNPS in GSK3β and SFRP4 genes

Gene	Chromosome	SNP ID	Allele	Minor Allele	Function
**GSK3β**	3q13.33	rs3732361	A/G	G	3′UTR
		rs3755557	T/A	A	Promoter
**SFRP4**	7p14.1	rs1052981	T/C	C	3′UTR
		rs1802073	A/C	C	Missense

UTR: untranslated regions

### Genotyping

**Figure 2:**
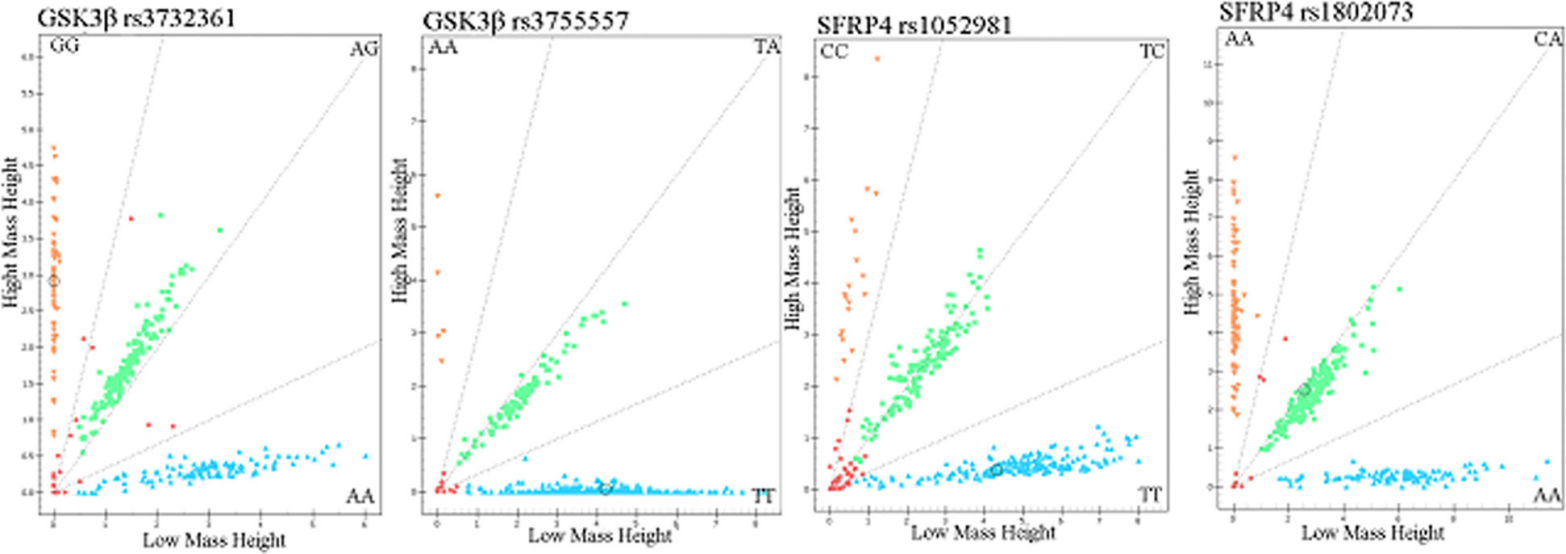
Call cluster plot of 4 variants genotyping in the GSK3β and SFRP4 genes by MALDI-ToF-MS The green plots represent heterozygous genotypes, yellow and blue plots represent major homozygous or minor homozygous genotypes in the call cluster plot of each variant, respectively. MALDI-ToF-MS: matrix-assisted laser desorption/ionization time-of-flight mass spectrometry.

Primers for polymerase chain reaction and sequencing were designed by Sequenom Assay Design 3.1 software (Sequenom, San Diego, CA, USA) following the manufacturer's instructions, shown in Table 6.The quality inspection of the sequencing primer was completed by matrix assisted laser desorption ionization time-of-flight mass spectrometry (MALDI-TOF). Mass ARRAY^®^platform (Sequenom Analyzer 4, Inc., San Diego, CA, USA) was used to analyse the 4 variants polymorphisms in the SFRP4 and GSK3β genes. The genotyping success rates for the 4SNPs were > 95%, respectively. The linkage disequilibrium analysis and Call Cluster Plot of the 4SNPs are presented in Figure 1 and Figure 2, respectively.

**Table 6:** List of PCR and sequencing primers of 4 variants in the SFRP4 and GSK3β genes

Gene	SNP ID	PCR Primer	Product length(bp)	Sequencing primer	Molecular weight
**SFRP4**	**rs1802073**	5′ ACGTTGGATGTTCTTCTTGGGACTGGCTGG3′	86	GTTTGGGAGCAGGAG	4713.1
		5′ ACGTTGGATGTCGTAGTAATCCCCCCAAAC 3′			
	**rs1052981**	5′ACGTTGGATGCAGAGCTGAAGTCATTGTAA 3′	132	AAGTCATTGTAAAAAAGACACATTATA	8307.5
		5′ACGTTGGATGTGTTTATGGTCTGCAGAAGG 3′			
**GSK3β**	**rs3732361**	5′ ACGTTGGATGCACTGACGTATCAAAACCTG3′	97	gGACGTATCAAAACCTGATACTATTA	7962.2
		5′ ACGTTGGATGCAAATGAGAGAGTGACAGAG3′			
	**rs3755557**	5′ACGTTGGATGGCAAGAGCCAGGTAATCTGA 3′	113	ccccATCTGATCAAATATAGGTCCTTT	8169.3
		5′ACGTTGGATGTCTACCTGCAGAGTCATCTC 3′			

### Statistical analysis

Statistical analysis was followed a prior study of our group [32]. Briefly, Shesis software platform (http://analysis.bio-x.cn/SHEsisMain.html) was used to analyse the Hardy-Weinberg equilibrium and haplotypes between the ONFH and control groups and their associations with the clinical phenotypes of ONFH. In addition, logistical regression analyses were performed to calculate the odds ratios (OR), 95% confidence intervals (CI), and corresponding *p*-values of each variants controlling for age and sex as covariates. The genetic models of dominant, recessive, and codominant were considered. SPSS10.0 software (X^2^ test) was used to analyse the association of the genes polymorphisms with clinical phenotypes of ONFH. A *p*-value of < 0.05 was considered statistically significant.
